# Berberrubine, a Metabolite of Berberine, Attenuates Intestinal Barrier Dysfunction in Inflammatory Bowel Disease by Inhibiting STAT3

**DOI:** 10.3390/ijms27146341

**Published:** 2026-07-16

**Authors:** Ziying Wang, Wanhong Zhu, Airu Ma, Jiaqi Chen, Jiawei Tian, Jiaying Zheng, Kang Wu, Xia Ding, Tiangong Lu

**Affiliations:** 1 School of Life Sciences, Beijing University of Chinese Medicine, Beijing 100029, China; 2 The First Clinical Medical College, Beijing University of Chinese Medicine, Beijing 100029, China; 3 School of Traditional Chinese Medicine, Beijing University of Chinese Medicine, Beijing 100029, China

**Keywords:** inflammatory bowel disease, berberine, berberrubine, intestinal barrier, tight junction, STAT3

## Abstract

Inflammatory bowel disease (IBD) is characterized by intestinal barrier dysfunction and excessive inflammation, in which STAT3 signaling plays a critical role. Berberine is clinically effective against colitis; its application is limited by low bioavailability and toxicity. Berberrubine, a major metabolite of berberine, exhibits improved pharmacological properties; however, its therapeutic potential in IBD remains unclear. The efficacy and mechanisms of berberine and berberrubine were evaluated in DSS-induced colitis mice, LPS-stimulated cells and intestinal organoids. *STAT3* knockout cell lines were generated using CRISPR-Cas9 to assess the role of STAT3 in mediating the pharmacological activities of both compounds. We found that berberrubine significantly alleviated colitis, reduced pro-inflammatory cytokines (IL-6 and TNF-α), and restored intestinal barrier integrity by upregulating ZO-1 and claudin-1 in vivo and in organoids, demonstrating superior efficacy and safety to berberine. Mechanistically, both compounds inhibited STAT3 activation and nuclear translocation. *STAT3* deficiency attenuated their anti-inflammatory, anti-tumor, and barrier-protective effects. Notably, berberrubine exhibited selective cytotoxicity to cancer cells over normal epithelial cells, suggesting a favorable therapeutic window. Collectively, berberrubine ameliorates intestinal barrier dysfunction and inflammation through inhibition of the STAT3 signaling pathway. Its superior efficacy and favorable safety profile compared to berberine support its potential as a novel therapeutic agent for IBD.

## 1. Introduction

Inflammatory bowel disease (IBD) is a chronic inflammatory condition arising from complex interactions between genetic susceptibility, environmental factors, and immune dysregulation. The main clinical subtypes are ulcerative colitis (UC) and Crohn’s disease (CD) [1]. Clinically, IBD follows a chronic, relapsing-remitting course, characterized by persistent symptoms including diarrhea, abdominal pain, and rectal bleeding. This disease also exhibits high recurrence rates and challenges in achieving a cure [2]. As a global health concern, IBD incidence is rising most rapidly in newly industrialized regions. In Asia, the incidence has reached 1.4 per 100,000 people and continues to climb, posing a substantial socioeconomic burden in China [3,4]. Beyond debilitating symptoms, persistent intestinal inflammation significantly increases the risk of colorectal cancer, with over 20% of patients with long-standing IBD developing this lethal malignancy within 30 years [5]. Current pharmacological therapies, including aminosalicylates, corticosteroids, and biologics, are limited by suboptimal efficacy, drug resistance, and adverse effects [6]. Therefore, identifying effective therapeutic strategies to interrupt this process is of critical clinical importance.

Disruption of intestinal barrier integrity and intestinal homeostasis is a critical initiating event in IBD and represents the initial trigger of inflammation [7]. The intestinal epithelial barrier is composed of specialized cells, such as goblet cells, enteroendocrine cells, Paneth cells and M cells. They are tightly interconnected and covered by a mucus layer secreted by goblet cells, forming the intestinal epithelial barrier [8]. This structure serves as the first line of immune defense, resisting pathogen invasion and producing antimicrobial peptides. Tight junctions (TJs) are essential components of the intestinal barrier, mainly comprising Zonula occludens-1 (ZO-1), occludin, and claudins. These proteins form a selective permeability barrier at the apical side of intestinal epithelial cells and participate in cell signal transduction, playing an important role in regulating intestine function and maintaining homeostasis [9]. Disruption of TJs allows toxins, antigens, and other harmful substances to penetrate the intestinal tissue, thereby activating the immune system and triggering inflammatory responses [10,11]. Restoring the intestinal epithelial barrier, therefore, represents a rational therapeutic direction for IBD.

Upon pathogen stimulation, activated inflammatory cascades lead to the secretion of pro-inflammatory cytokines such as interleukin-6 (IL-6), tumor necrosis factor-α (TNF-α), and interleukin-1β (IL-1β), which in turn activate multiple signaling pathways during the early stages of IBD [12]. Among these, the JAK-STAT pathway plays a critical role in linking barrier dysfunction to sustained inflammation in IBD. Upon stimulation by IL-6 and TNF-α, phosphorylated STAT3 (p-STAT3) translocates to the nucleus, aggravating the mucosal inflammatory response and damaging the intestinal epithelial barrier [13]. Indirectly, IL-6 upregulates integrin expression to activate FAK, which enhances cell-ECM adhesion, contributing to a coordinated network for comprehensive STAT3 activation [14]. Uncontrolled inflammatory responses further recruit macrophages and neutrophils, leading to tissue damage and ulcer formation [15], and even cause intestinal fibrosis in advanced stages of IBD [16]. Consequently, inhibition of STAT3 signaling represents a promising therapeutic strategy for IBD.

Traditional Chinese medicine (TCM) has demonstrated unique clinical advantages in treating IBD. Berberine, an isoquinoline alkaloid extracted from various TCM plants of the *Berberis* genus such as *Coptis chinensis* and *Phellodendron amurense*, have been traditionally used to treat intestinal disorders including dysentery and enteritis [17,18]. Berberine exerts broad pharmacological effects, including antibacterial, anti-inflammatory, anti-tumor, and metabolic regulatory activities [19,20]. Clinically, berberine is predominantly administered as an adjunct to conventional therapies, particularly in combination with 5-aminosalicylic acid [21,22]. Clinical studies have demonstrated that berberine effectively alleviates diarrhea and abdominal pain, reduces inflammatory markers, and improves clinical outcomes in IBD patients [23,24]. These findings support its therapeutic value in the management of intestinal inflammation.

Despite these promising biological activities, berberine faces several challenges. Its therapeutic window is narrow, and long-term administration at high doses may cause adverse reactions including hepatotoxicity and gastrointestinal disturbances [19,25,26]. Moreover, berberine is a substrate of P-glycoprotein (P-gp), which actively pumps it back into the intestinal lumen, resulting in low oral bioavailability (less than 1%) [27]. These pharmacokinetic limitations suggest that the actual therapeutic effects of berberine in vivo may be mediated by its active metabolites of berberine [28].

**Figure 1:**
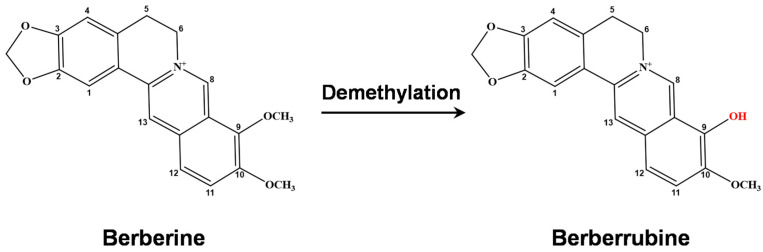
The structural formula and metabolic relationship between berberine and berberrubine.

Berberrubine, a major metabolite of berberine formed via demethylation at the C-9 position (Figure 1), has demonstrated superior efficacy in ameliorating intestinal injury, reducing inflammatory factors levels, and restoring mucosal barrier function at lower doses compared to its parent compound [29]. The hydroxyl group at the C-9 position also facilitates structural modification, potentially enhancing its binding affinity and target selectivity [30]. Berberrubine has been reported to ameliorate DSS-induced colitis by preserving intestinal barrier integrity and modulating inflammatory responses [31]. However, research on berberrubine in the context of IBD remains limited, with insufficient understanding of its toxicological profile and the molecular mechanism underlying its pharmacological effects.

Herein, we evaluated the therapeutic effects and the mechanisms of berberine and its metabolite berberrubine in IBD. In a DSS-induced mouse model, berberrubine alleviated DSS-induced colitis by restoring intestinal mucosal barrier and inhibiting inflammatory responses, demonstrating superior efficacy compared to berberine at the same dosage. Mechanistically, berberrubine promoted apoptosis, upregulated the expression of tight junction proteins and cell migration by inhibiting the STAT3 pathway. This led to decreased expression of pro-inflammatory factors and preserved intestinal barrier function. Together, our findings highlight berberrubine as a key active metabolite of berberine with therapeutic potential in IBD. This work provides new insights for optimizing berberine-based therapy and lays an experimental foundation for the structural development of berberine derivatives.

## 2. Results and Discussion

### 2.1. Berberine and Berberrubine Alleviate DSS-Induced Colitis in Mice

**Figure 2:**
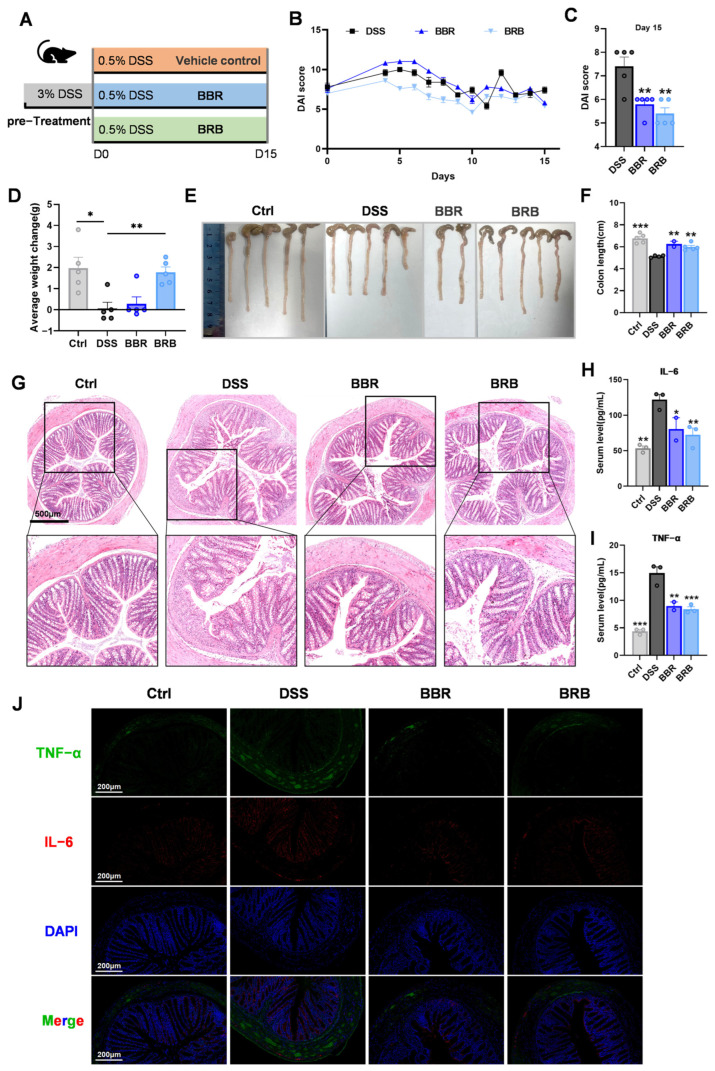
Berberine and berberrubine alleviate DSS-induced colitis in mice. (**A**) Time flow chart. (**B**–**F**) Disease activity index (DAI) scores (**B**,**C**), average body weight (**D**), colon image (**E**) and colon length (**F**) of mice in each group. (**G**) H&E staining of colon tissue, Scale bar: 500 μm. (**H**,**I**). Serum level of IL-6 (**H**) and TNF-α (**I**) measured by ELISA. All data are shown as mean ± SEM: * *p* < 0.05, ** *p* < 0.01, *** *p* < 0.001 (one-way ANOVA) vs. DSS-induced group. (**J**). Immunofluorescence staining of IL-6 and TNF-α in colon tissue. Scale bar: 200 μm.

To evaluate the therapeutic effect of berberrubine, a DSS-induced mouse model of colitis was established (Figure 2A). After 7 days of DSS induction, mice in the model group exhibited a typical colitis phenotype, including increased disease activity index (DAI), decreased body weight, and shorter colon length compared to control mice (Figure 2B–F). Treatment with berberine or berberrubine significantly alleviated these disease manifestations. Pathological analysis confirmed that the colon of mice in the model group displayed features of acute ulcerative colitis, including inflammatory cell infiltration, pronounced mucosal damage, deformation of intestinal epithelial cells, extensive colonic ulcers, and crypt damage or loss. In contrast, berberine or berberrubine remarkably ameliorated the pathological condition of the colon tissue (Figure 2G).

Given that IBD onset is often accompanied by abnormal immune activation, we measured the serum and tissue level of pro-inflammatory cytokines. Consistently, berberine and berberrubine remarkably reduced DSS-induced IL-6 and TNF-α elevation (Figure 2H,I). Berberrubine treatment substantially reduced the serum levels of IL-6 and TNF-α in mice to 59% and 56%, respectively, compared to the model group. Furthermore, immunofluorescence staining at the site of inflammation revealed a similar result in the expression levels of IL-6 and TNF-α (Figure 2J). Notably, these effects approached normal control levels.

The safety of the drugs was assessed based on changes in body weight, survival rates, and general appearance. Survival analysis revealed that berberine treatment resulted in earlier mortality and a greater total number of deaths than berberrubine treatment (Supplementary Figure S1A). During the experimental period, mice in the berberine group exhibited a greater degree of body weight loss (Supplementary Figure S1B). Berberine-treated mice exhibited multiple symptoms of drug toxicity, such as lethargy, sparse and lackluster fur, abdominal distension, and markedly decreased feeding and drinking. Necropsy further revealed intestinal obstruction and ascites. Collectively, these observations demonstrate that berberine possesses greater toxicity than berberrubine.

### 2.2. Berberine and Berberrubine Restore Cell Proliferation and Promote Apoptosis in Intestinal Epithelial Cells

**Figure 3:**
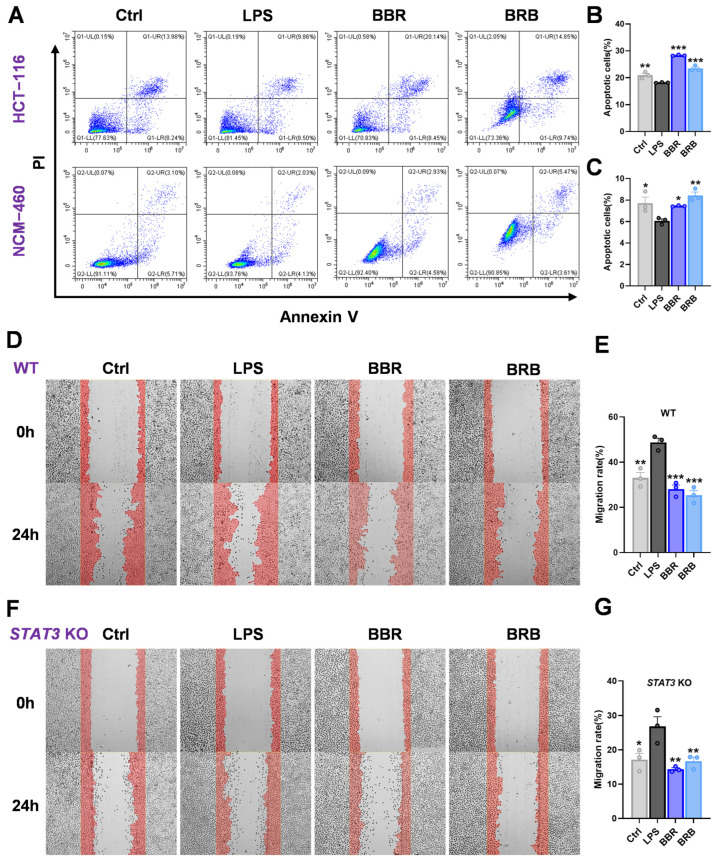
Berberine and berberrubine inhibit proliferation and promote apoptosis. (**A**) HCT-116 and NCM-460 cells were exposed to indicated compounds for 48 h. Cell apoptosis were stained with Annexin V/PI and assessed by flow cytometry. (**B**,**C**) Quantification of apoptosis in HCT-116 (**B**) and NCM-460 (**C**) cells. Cells heating to 55 °C water bath incubation for 5–10 min was served as the positive control (Supplementary Figure S1G). (**D**–**G**) The effect of berberine and berberrubine on cell migration ability was evaluated through cell scratch assays for (**D**) NCM-460 WT and (**F**) *STAT3* KO cell line. Corresponding quantitative analyses are shown in panels E and G. All data are shown as mean ± SEM: * *p* < 0.05, ** *p* < 0.01, *** *p* < 0.001 (one-way ANOVA) vs. LPS group.

Dysregulated proliferation and impaired apoptosis of intestinal epithelial cells are key features of IBD-associated mucosal inflammation and tumorigenesis [32]. In normal human intestinal epithelial NCM-460 cells, inflammatory stimulation suppressed cell apoptosis (Figure 3A,B). Treatment with berberine or berberrubine reversed this inhibition, restoring apoptosis to near basal levels. In HCT-116 colorectal cancer cells, berberine or berberrubine promoted apoptosis and exerted cytotoxic effect, demonstrating significant anti-tumor efficacy (Figure 3A,C).

In cells, inflammatory stimulation upregulated the expression of inflammation-related signaling pathway, including IL-6/STAT3, leading to abnormal proliferation. This uncontrollable proliferation of intestinal epithelial cells may drive malignant transformation and ultimately promote the progression from inflammation to cancer [33]. Scratch assay was performed in NCM-460 cells (Figure 3D,E). Berberine or berberrubine effectively inhibited this process, restoring the migration capacity to near basal levels.

To further investigate the mechanism, we generated *STAT3* knockout (KO) cell lines using CRISPR-Cas9 genome editing system (Supplementary Figure S1C–F). Comparison of cell migration between wild-type and *STAT3* KO cells revealed that the migration rate of WT cells was higher than that of *STAT3* KO cells, indicating that loss of *STAT3* weakened the migration and repair capacity of normal intestinal epithelial cells, thereby hindering the healing of damaged epithelium (Figure 3F,G). Notably, berberine or berberrubine failed to effectively restore LPS-induced cell migration in *STAT3* KO cells, suggesting that STAT3 expression plays a critical role in mediating the protective effects of these compounds. Collectively, these data demonstrate that berberine and berberrubine exert their protective effects by regulating the STAT3 signaling pathway.

### 2.3. Berberine and Berberrubine Promote Intestinal Mucosal Integrity

**Figure 4:**
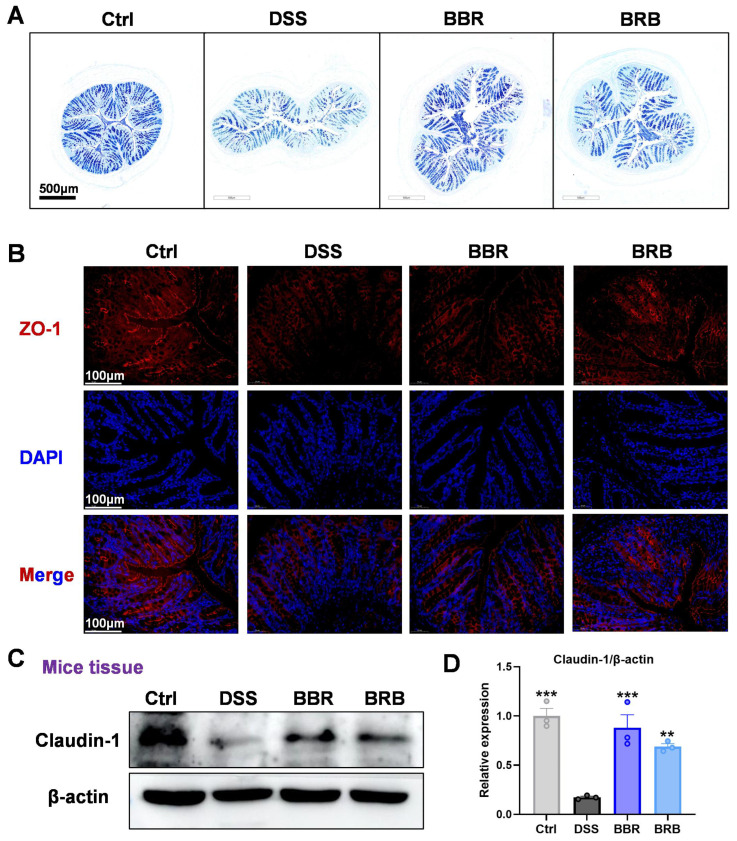
Berberine and berberrubine promote intestinal mucosa integrity. (**A**) AB-PAS staining of colon tissue. Scale bar: 500 μm. (**B**) Immunofluorescence staining of ZO-1 in colon tissue. Scale bar: 100 μm. (**C**) Western blot analysis of Claudin-1 proteins expression in colon tissue. (**D**) Quantification of Claudin-1, mean ± SEM: ** *p* < 0.01, *** *p* < 0.001 (one-way ANOVA) vs. DSS-induced group.

AB-PAS staining was performed to detect the expression of mucin in mice colon. In DSS-induced mice, the number of goblet cells and the amount of mucin produced were significantly reduced, accompanied by loosening of the intestinal wall structure and loss of crypt structure. Berberine or berberrubine treatment reversed these abnormalities (Figure 4A). ZO-1, a key regulator of tight junction function, was revealed with high expression in normal mice. In contrast, ZO-1 expression was remarkably reduced in DSS-treated colitis mice. Following berberine or berberrubine treatment, the expression level of ZO-1 in the colon was recovered. Especially, colon of berberrubine-treated mice showed higher expression levels than that of berberine group, revealing berberrubine possesses better efficacy in improving the intestinal barrier integrity (Figure 4B).

We further detected the expression of intestinal mucosal integrity protein Claudin-1. DSS-induced colitis severely reduced Claudin-1 expression in colonic tissues by nearly 85% (Figure 4C,D). In contrast, berberine or berberrubine treatment significantly restored Claudin-1 expression to 88% and 69% of control levels, respectively. These results confirm that both compounds restore Claudin-1 expression in damaged intestinal epithelial cells and efficiently repair the intestinal tight junction function.

### 2.4. Berberine and Berberrubine Restore the Barrier Function of Intestinal Organoids

**Figure 5:**
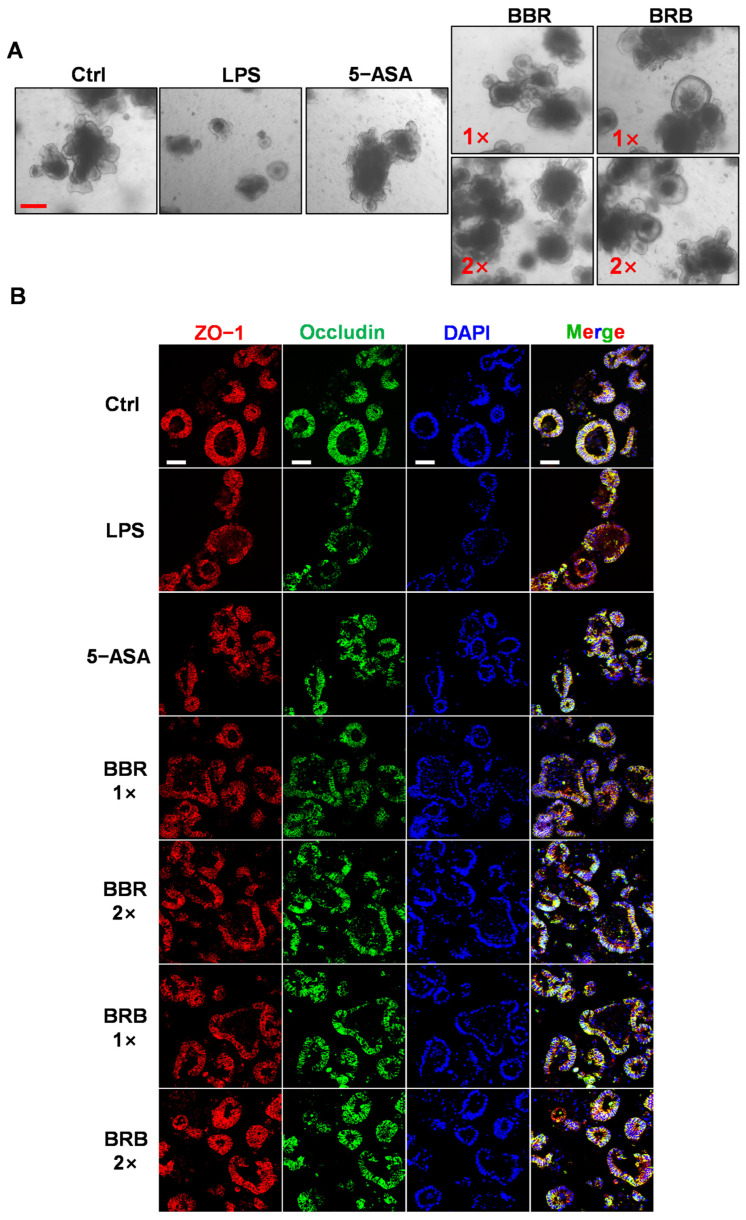
Berberine and berberrubine restore the barrier function of intestinal organoids. (**A**) Bright-field images of intestinal organoids following 24 h treatment with 1 mM 5-ASA, berberine (1× IC_50_ and 2× IC_50_), or berberrubine (1× IC_50_ and 2× IC_50_) after LPS stimulation. Scale bar: 100 μm. (**B**) Immunofluorescence staining of ZO-1 and Occludin of organoid. Organoids were exposed to 100 μg/mL LPS for 24 h, followed by treatment with 1 mM 5-ASA, berberine (1× IC_50_ and 2× IC_50)_, or berberrubine (1× IC_50_ and 2× IC_50_) for 24 h. Scale bar: 50 μm.

Intestinal organoids recapitulate key structural and functional features of the native intestinal epithelium and have been recognized as a valuable platform for drug discovery in IBD [34]. To further investigate the effects of BBR and BRB on intestinal barrier function, we isolated intestinal crypts from mice and established an LPS-induced injury model using intestinal organoids (Figure 5A and Figure S2). 5-Aminosalicylic acid (5-ASA), a first-line drug for IBD, was included as a positive control.

LPS treatment remarkably disrupted the epithelial cell arrangement and significantly diminished the expression of tight junction proteins ZO-1 and Occludin, indicating increased gut permeability and compromised barrier integrity (Figure 5B). Treatment with 5-ASA at 1 mM effectively reversed these alterations. Interestingly, while 5-ASA at 1 mM showed clear protective effects, higher concentrations (5 mM and 10 mM) induced organoid shrinkage and cell death, suggesting potential cytotoxicity (Supplementary Figure S3). Consistent with the observation from in vivo and cell-based findings, BBR and BRB restored barrier function in a concentration-dependent manner. Both compounds exhibit more pronounced effects at 2× IC_50_ than at 1× IC_50_. The protective effects of the two compounds were comparable to those of 5-ASA.

### 2.5. Berberine and Berberrubine Inhibit STAT3 Signaling Pathway In Vitro

**Figure 6:**
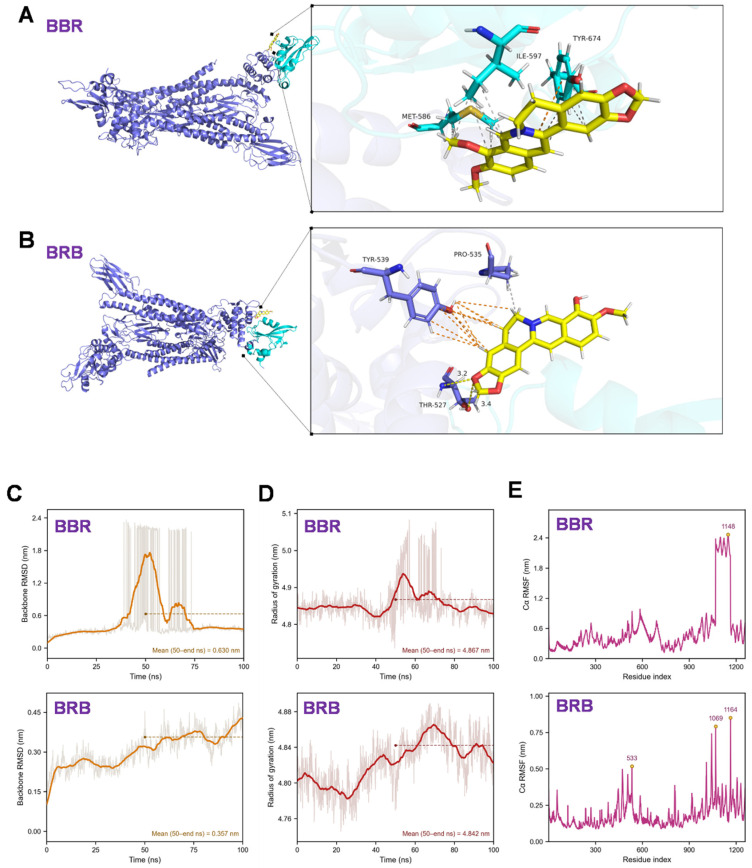
Molecular docking and dynamics of STAT3 SH2 domain with berberrubine and berberine. (**A**,**B**) Molecular docking of STAT3 SH2 domain with berberrubine and berberine. Proteins are shown in purple, the SH2 domain in cyan, and the small molecule as yellow sticks. Gray dashed lines represent van der Waals/hydrophobic contacts, orange dashed lines represent aromatic proximity interactions, and yellow dashed lines represent hydrogen-bonding/polar contacts. (**C**) Root mean square deviation (RMSD). (**D**) Radius of gyration (RG). (**E**) Cα Root mean square fluctuation (Cα RMSF).

Molecular docking was first performed to predict the interaction between berberine or berberrubine and STAT3 (Figure 6A,B). Berberrubine showed a binding energy of −11.36 kcal/mol, with polar close contacts to Thr527 within the SH2 domain of chain A (distances of approximately 3.20 Å and 3.42 Å). Berberine exhibited a binding energy of −12.12 kcal/mol, with key interactions involving Ile 597, Met 586 and Tyr 674 in the STAT3 SH2 domain, though no stable polar contacts within 3.5 Å were observed yet. These results reveal the high affinity of both berberine and berberrubine for STAT3. To evaluate dynamic stability of the protein-ligand complexes, molecular dynamics simulations were performed (Figure 6A–C and Figure S4A–D). Berberrubine exhibited minimal structural fluctuations, with a mean backbone RMSD 0.357 nm over 50–100 ns (Figure 6C). In contrast, berberine exhibited higher conformational fluctuations, with mean backbone RMSD 0.631 nm over the same interval, despite a lower final frame RMSD of 0.358 nm. Both systems remained compact global structures (Rg 4.8–4.9 nm) and low hydrogen bond frequencies (0.055 and 0.038 for berberrubine and berberine, respectively) (Figure 6D and Figure S4A). Moreover, berberine induced more pronounced localized dynamics with the maximum Cα RMSF of 2.463 nm, compared to that of 0.852 nm for berberrubine, suggesting that berberrubine binds in a more stable, low-perturbation manner (Figure 6E).

**Figure 7:**
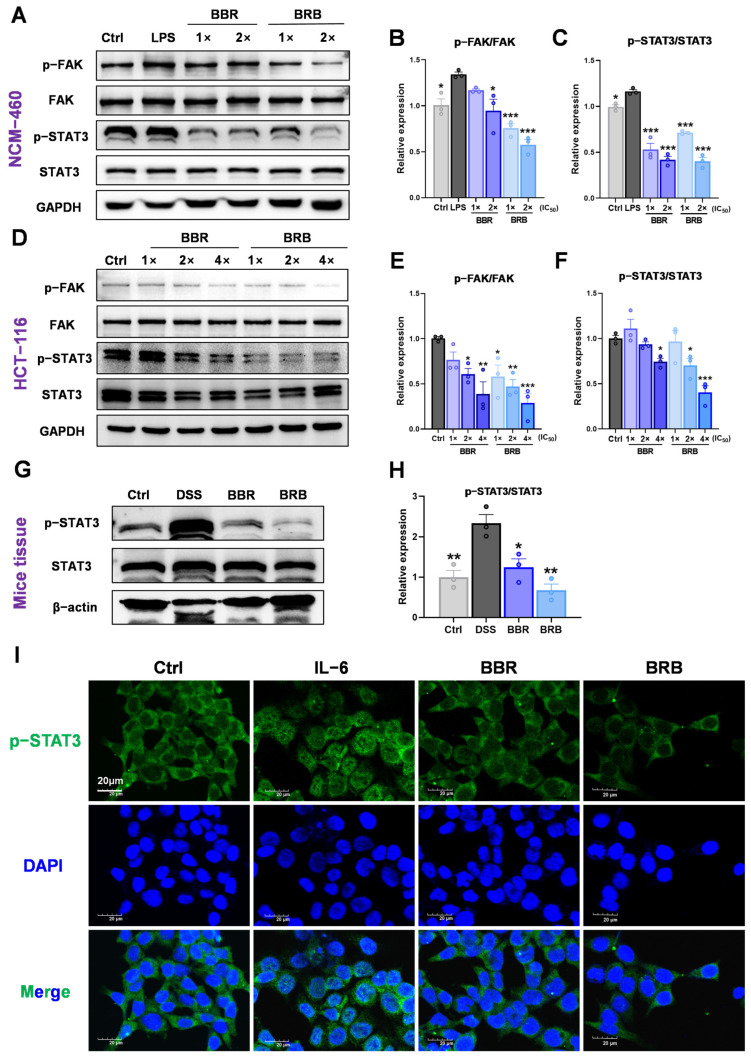
Berberine and berberrubine inhibit STAT3 signaling pathway in vitro. (**A**–**F**) Western blot analysis of phosphorylated STAT3 and phosphorylated FAK in NCM-460 (**A**) and HCT-116 (**D**) cells treated with berberine and berberrubine. Corresponding quantitative analyses are shown in panels (**B**,**C**,**E**,**F**). (**G**) Western blot analysis of p-STAT3 in colon tissue. (**H**) Quantification of p-STAT3. (**I**) Immunofluorescence staining of p-STAT3 nuclear translocation. Scale bar: 20 μm. All data are shown as mean ± SEM: * *p* < 0.05, ** *p* < 0.01, *** *p* < 0.001 (one-way ANOVA) vs. LPS-induced group or DSS-induced group.

We next examined the regulatory effects of berberine and berberrubine on the STAT3 signaling pathway. In LPS-induced NCM-460 inflammatory cells, p-FAK and p-STAT3 were markedly increased upon LPS stimulation, reaching 134% and 116% of control levels, respectively. Berberine and berberrubine effectively suppressed FAK and STAT3 activation in a dose-dependent manner (Figure 7A–C). Notably, berberrubine exhibited a more robust inhibitory effect on p-FAK compared to berberine. It reduced p-FAK expression to 76% of control levels at IC_50_ and further to 58% at higher concentration (2× IC_50_). In contrast, berberine showed no significant suppression at IC_50_ and achieved only 5% inhibition compared to control at 2× IC_50_. Both compounds exhibited even greater inhibition on p-STAT3. Berberine showed superior efficacy at the lower concentration, but this difference diminished at the higher concentration, where both achieved comparable inhibition, with inhibition rates of 42% and 40%, respectively. Consistently, both compounds exhibited concentration-dependent inhibition of p-FAK and p-STAT3 activation in the colon tumor HCT-116 (Figure 7D–F) and LS513 cell lines (Supplementary Figure S5A,B). In vivo experiments confirmed these findings. DSS-induced colitis remarkably activated p-STAT3 expression in colonic tissues by nearly 233% (Figure 7G,H), while berberine or berberrubine treatment reduced the expression level of p-STAT3 to 88% and 69% of the model group levels, respectively.

Nuclear translocation of p-STAT3 is essential for its transcriptional activity. We investigated whether the reduced p-STAT3 levels induced by the compounds reflect impaired nuclear translocation in cells. Immunofluorescence staining of p-STAT3 showed that IL-6 increased nuclear p-STAT3 localization (Figure 7I). Both compounds diminished p-STAT3 fluorescence intensity in HCT-116 cells. Notably, berberrubine demonstrated a stronger inhibitory effect on STAT3 phosphorylation and subsequent nuclear activation compared to berberine.

### 2.6. STAT3 Is Required for the Protective Effects of Berberine and Berberrubine

**Figure 8:**
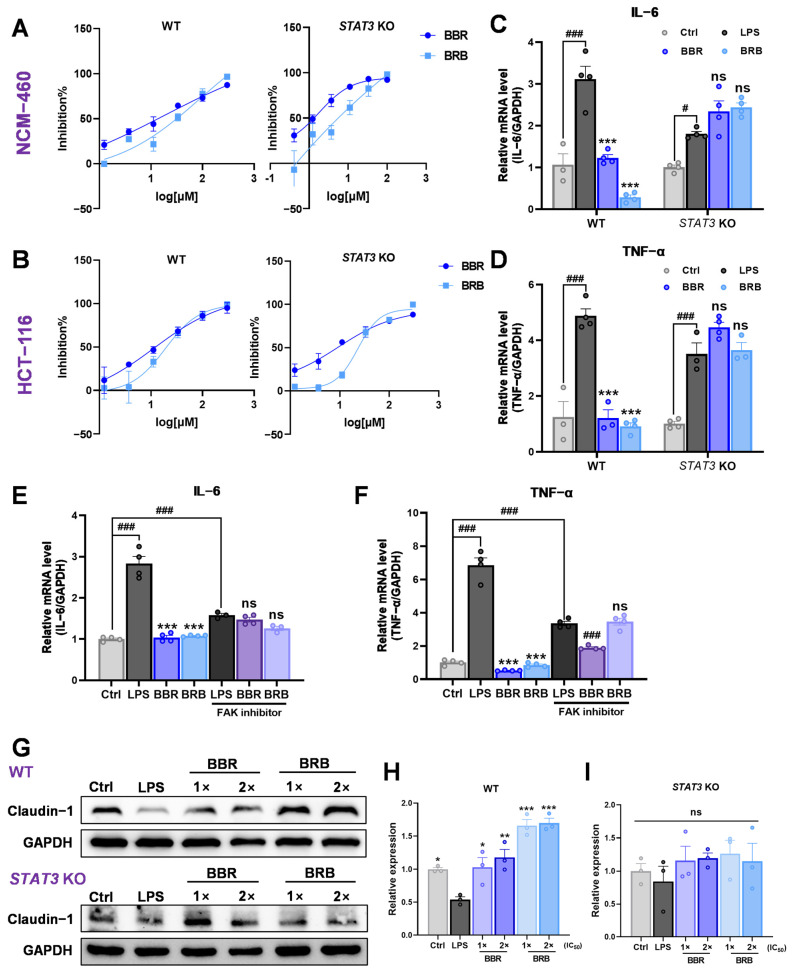
Berberine and berberrubine reverse the LPS-induced inflammation and reduction in tight junction proteins by inhibiting the JAK-STAT3 signaling pathway. (**A**,**B**) Growth inhibition curves of berberine and berberrubine in WT versus *STAT3* KO cell lines: (**A**) NCM-460 and (**B**) HCT-116. (**C**,**D**) The mRNA levels of IL-6 (**C**) and TNF-α (**D**) in NCM-460 WT versus *STAT3* KO cell lines treated with berberine and berberrubine. (**E**,**F**) The mRNA levels of IL-6 (**E**) and TNF-α (**F**) in NCM-460 WT cell lines treated with or without FAK inhibitor PF-573228 simultaneously before berberine and berberrubine administration. (**G**) Western blot analysis of Claudin-1 in NCM-460 WT versus *STAT3* KO cell lines treated with berberine and berberrubine for 24 h. Corresponding quantitative analyses are shown in panels (**H**,**I**), All data are shown as mean ± SEM: ns (not significant), * *p* < 0.05, ** *p* < 0.01, *** *p* < 0.001 (one-way ANOVA) vs. LPS-induced group, and # *p* < 0.05, ### *p* < 0.001 (one-way ANOVA) vs. control group.

To determine whether STAT3 is functionally required for the protective effects of berberine and berberrubine, we evaluated the cytotoxicity of both compounds using two established *STAT3* KO cell models (Table 1). Overall, berberine exhibited lower IC_50_ values than berberrubine in both NCM-460 normal cells and HCT-116 cancer cells, indicating a greater anti-proliferative effect. Specifically, berberrubine showed selective cytotoxicity to cancer cells compared to normal colon epithelial cells, whereas berberine was three times more potent in normal epithelial cells (IC_50_ = 4.3 μM) than in cancer cells (IC_50_ = 12.3 μM). Moreover, knockout of *STAT3* altered the sensitivity of both compounds towards cells, indicating that STAT3 expression is closely associated with the cytotoxicity of both berberine and berberrubine (Figure 8A,B).

We next examined whether STAT3 is required for the anti-inflammatory effects of both compounds (Figure 8C,D). LPS treatment significantly increased IL-6 and TNF-α mRNA levels by 3.1- and 4.9-fold in NCM-460 cells, respectively. Treatment with berberine or berberrubine significantly downregulated both inflammatory cytokines and restored their levels to nearly normal. In contrast, loss of *STAT3* alone attenuated the LPS-induced elevation of IL-6 and TNF-α, though their levels remained higher than those in untreated controls. Moreover, both compounds failed to further reverse the upregulated IL-6 and TNF-α in *STAT3* KO cells, suggesting that the anti-inflammatory effects of berberine and berberrubine are closely dependent on STAT3.

To further investigate the role of FAK in mediating the anti-inflammatory effects of berberine and berberrubine, cells were pretreated with FAK inhibitor (PF-573228) prior to LPS and compound treatment (Figure 8E,F and Figure S5C). FAK inhibition alone significantly reversed the LPS-induced cytokine expression (IL-6 and TNF-α). Especially, under FAK inhibition, neither berberine nor berberrubine further reduced IL-6 and TNF-α levels beyond those observed with FAK inhibitor alone. These results indicate that the anti-inflammatory effects of both compounds are FAK-dependent.

Given that STAT3 expression closely affects the cytotoxic and anti-inflammatory effects of both compounds, we next examined whether STAT3 is also required for their protective effects on intestinal barrier integrity. The expression of claudin-1 was assessed in NCM-460 cells. LPS-induced inflammation significantly reduced claudin-1 expression, which was reversed by treatment with berberine (Figure 8G). Berberrubine exhibited a better effect, restoring claudin-1 expression and further increasing it to 1.66-fold of control levels at the IC_50_ concentration. Interestingly, *STAT3* deficiency compromised the rescue effect of both compounds (Figure 8H,I). Neither compound significantly rescued the LPS-induced suppression of claudin-1 in *STAT3* KO cells, suggesting that berberine and berberrubine regulate claudin-1 expression through STAT3-dependent mechanisms.

Collectively, these data demonstrate that FAK/STAT3 signaling mediates the cytotoxic, anti-inflammatory, and barrier-protective effects of berberine and berberrubine. Moreover, berberrubine exhibits greater potency and selectivity, particularly in restoring intestinal barrier function.

### 2.7. Discussion

In this study, we combined in vivo and in vitro approaches with CRISPR-Cas9-mediated *STAT3* knockout to compare the pharmacodynamics and mechanisms of berberine and its major metabolite berberrubine in IBD. For the first time, we demonstrate that berberrubine exhibits superior efficacy and better safety than berberine at equivalent doses in alleviating DSS-induced colitis. Mechanistically, berberrubine restores intestinal barrier integrity and suppresses inflammation by inhibiting STAT3 activation and nuclear translocation. STAT3 knockout further confirmed that STAT3 is critically required for the protective effects of both compounds. Importantly, berberrubine showed selective cytotoxicity to cancer cells over normal epithelial cells.

Intestinal barrier dysfunction and excessive inflammation are hallmarks of IBD and critical drivers of disease progression [35,36,37]. Disruption of tight junction proteins, including ZO-1, claudins, and occludins, increases intestinal permeability and allows harmful substances such as toxins and antigens to penetrate intestinal tissue, thereby activating the immune system and triggering inflammatory responses, even increasing the risk of colorectal cancer [9,38]. Overactivation of inflammatory pathways leads to massive release of pro-inflammatory cytokines such as IL-6 and TNF-α, which further aggravates epithelial damage and establishes a vicious cycle of inflammation [8]. Consistently, we observed that DSS-induced colitis markedly reduced ZO-1 and claudin-1 expression and elevated serum IL-6 and TNF-α levels. Importantly, we demonstrate that berberrubine treatment effectively restores tight junction proteins and suppresses inflammatory cytokine production to near-normal levels, notably outperforming berberine at equivalent doses. While berberine has been reported to exert both anti-inflammatory and barrier-protective effects [24], our study provides the first direct comparison showing that its metabolite berberrubine exhibits superior efficacy. This raises the possibility that the therapeutic effects of berberine in IBD may be partially attributed to its in vivo conversion to berberrubine. This is further supported by organoid data. From our observation, both compounds restored tight junction proteins upon LPS injury with efficacy comparable to 5-ASA. Notably, while 5-ASA at 5–10 mM, a concentration range previously used in organoid studies [39,40,41], induced evident cytotoxicity, berberrubine maintained barrier protection without such toxicity at the tested concentrations. Mechanistically, previous studies have established that berberrubine ameliorates DSS-induced colitis, restores tight junction proteins, and reduces inflammatory cytokines [31]. However, the molecular mechanism underlying these protective effects remained unclear.

Here, we identify STAT3 as a critical mediator. Specifically, STAT3 plays an important role in linking inflammation to barrier dysfunction in IBD [16]. Previous studies have established that STAT3 activation promotes the expression of various pro-inflammatory cytokines and impairs intestinal epithelial integrity [13,19]. Pharmacological inhibition of STAT3 ameliorates colitis in experimental models [42,43]. Our molecular docking analysis revealed that both berberine and berberrubine bind directly to STAT3. Functional studies further confirmed that both compounds inhibit STAT3 phosphorylation and nuclear translocation, leading to reduced IL-6 and TNF-α production and restored claudin-1 expression. The use of *STAT3* knockout cell models further confirmed that these protective effects of both compounds are STAT3-dependent.

Berberine has demonstrated favorable therapeutic efficacy in treating IBD, effectively ameliorating intestinal inflammation and alleviating clinical symptoms such as abdominal pain, diarrhea, and hematochezia [17,24,44]. However, its clinical application is hindered by low bioavailability and potential toxicity [27]. Berberrubine, a major metabolite of berberine, possesses improved pharmacokinetic properties, allowing more efficient intestinal absorption [29]. While berberine has been shown to suppress STAT3 activation in various disease models [45,46], we provide the first evidence that berberrubine exhibits more potent inhibition of STAT3 signaling, particularly at lower concentrations. In addition, berberrubine also showed selective cytotoxicity to cancer cells over normal epithelial cells, highlighting the translational potential of berberrubine as a more effective and safer alternative to berberine. Berberrubine exhibits the highest tissue distribution in the liver and limited penetration across the blood–brain barrier, which fundamentally circumvents potential neurotoxicity [47]. Furthermore, berberrubine exists in different protonation states depending on the pH of the microenvironment. It adopts an enol form (protonated state) under acidic conditions, whereas it converts to a quinone-like form (deprotonated state) under alkaline conditions [29]. This pH-dependent absorption and metabolic characteristic may further facilitate its targeted action on inflamed lesions in inflammatory bowel disease.

Several limitations of this study should be acknowledged. First, while we used berberine as a positive control for horizontal comparison, a standard-of-care drug such as 5-ASA was not included as an additional positive control in the DSS-induced colitis model. Second, only a single dose (50 mg/kg/day) of each compound was tested in vivo; dose–response studies would provide more comprehensive information on the pharmacological profile of berberrubine.

Finally, IBD pathogenesis involves complex immune-epithelial interactions. The intestinal epithelial barrier controls microbial translocation and directs mucosal immune responses, and its dysfunction triggers immune cell activation and chronic inflammation [11]. STAT3-inducing cytokines such as IL-6 and IL-22, produced by both immune cells and epithelial cells, control epithelial proliferation, survival, and barrier integrity, and dysregulation of this signaling axis contributes to IBD pathogenesis [16]. While our study focused on epithelial STAT3 as the primary mechanism, further studies on investigating whether berberrubine modulates immune cell function and immune-epithelial crosstalk will help to fully elucidate its therapeutic mechanism in IBD.

## 3. Material and Methods

### 3.1. Reagents

Berberine hydrochloride (S01577) was purchased from Dilger Medical Technology Co., Ltd. (Nanjing, China). Berberrubine (BD328621) was obtained from Bide Pharmatech (Shanghai, China). Berberine hydrochloride tablets were provided by Northeast Pharmaceutical Group (Shenyang, China). Dextran sulfate sodium (DSS, 02160110-CF) was purchased from MP Biomedicals (Irvine, CA, USA). Fecal occult blood detection kit (TC0511) was purchased from Leagene Biotechnology Co., Ltd. (Beijing, China). Mouse tumor necrosis factor-α ELISA kit (E-EL-M3063) and mouse interleukin-6 ELISA kit (E-EL-M0044) were obtained from Elabscience Biotechnology Co., Ltd. (Wuhan, China). Lipopolysaccharide (LPS, AL8880) was obtained from Acmec Biochemical Technology Co., Ltd. (Shanghai, China). 5-Aminosalicylic acid (A79809) was purchased from Sigma-Aldrich (St. Louis, MO, USA). Thiazolyl blue powder (M2128) was purchased from Sigma, Merck KGaA (Darmstadt, Germany). Reagents for cell culture and Western blot were obtained from Solarbio (Beijing, China). Pharmingen™ FITC Annexin V Apoptosis Detection Kit I (556547) was obtained from Becton, Dickinson and Company (Franklin Lakes, NJ, USA). qPCR reagents were purchased from Novoprotein Scientific Inc. (Suzhou, China). Primary antibodies for GAPDH (60004) and secondary antibodies were obtained from Proteintech (Wuhan, China). Antibodies for p-STAT3 (Tyr705, #9145) and STAT3 (#9139) were purchased from Cell Signaling Technology (Danvers, MA, USA). Antibody for Claudin-1 (56168) was purchased from Signalway Antibody Co., Ltd. (Nanjing, China).

### 3.2. Animal Study

Female C57BL/6 mice (6 weeks old) were supplied by SPF Biotechnology Co., Ltd. (Beijing, China). Mice were housed in a specific pathogen-free room under controlled environmental conditions (25 °C, relative humidity 40–70%) with a 12 h light/dark cycle. Sterilized cages, bedding, drinking water, and feed were provided, and animals had free access to food and water. All experimental procedures and animal care were approved by the Experimental Animal Ethics Committee of Beijing University of Chinese Medicine (Approval No. BUCM-2023122101-4226).

The mice were administered 3% DSS in drinking water for 7 days and administration after that, while control mice received normal drinking water to establish colitis model. Body weight, stool consistency, and fecal occult blood were recorded every three days during the experiment. Each parameter was scored on a scale of 0–4 according to the severity, and the disease activity index (DAI) score was calculated as the sum of the three parameters (Table 2). Berberine or berberrubine (50 mg/kg/day) or vehicle solution was administrated daily by oral gavage, with 0.5% DSS continuously provided in drinking water to maintain the colitis condition. At the endpoint of the experiment, the blood, colon tissue was collected for further analysis.

### 3.3. Cell Culture and Model Establishment

The NCM-460 normal human colon epithelial cell line was purchased from Shanghai Enzyme Research Biotechnology Co., Ltd. (Shanghai, China). The HCT-116 colorectal cancer cell line was obtained from the Cell Resource Center, Peking Union Medical College. Cells were cultured with RPMI-1640 supplemented with 10% heat-inactivated fetal bovine serum (Gibco™, A5256701, Waltham, MA, USA) and 1% penicillin-streptomycin solution in a humidified atmosphere of 37 °C with 5% CO_2_. To establish an inflammation cell model, cells were serum-starved and stimulated with 50 μg/mL lipopolysaccharide (LPS) for 24 h.

### 3.4. CRISPR-Cas9 Mediated Gene Editing

Cas9 protein v2 (A36497), Lipofectamine™ CRISPRMAX™ Cas9 (CMAX00001), TrueGuide™ Synthetic sgRNA sequences of STAT3 (A35533, CATTCGACTCTTGCAGGAAG) were designed and purchased from Invitrogen™, ThermoFisher Scientific (Carlsbad, CA, USA). Gene editing was carried out following the method described previously [48]. Briefly, after transfection, mixed cloned cells were collected for single-cell cloning culture, and STAT3 expression was evaluated by Western blotting (Supplemental Figure S1C,E). Clones lacking STAT3 expression were further confirmed by Sanger sequencing. Homozygous deletion on *STAT3* gene sequence was confirmed in NCM-460 *STAT3* KO cells (1 bp insertion) and HCT-116 *STAT3* KO cells (7 bp deletion) (Supplemental Figure S1D,F).

### 3.5. MTT Assay

Cells were seeded at a density of 5000 cells per well in 96-well plates overnight. Cells were treated with berberine or berberrubine for 72 h. MTT solution (5 mg/mL) was added, and the formazan crystals were dissolved in DMSO following by 4 h incubation. Absorbance was measured at 570 nm using a microplate reader (SpectraMax^®^ i3x, Molecular Devices, San Jose, CA, USA), and the 50% inhibitory concentration (IC_50_) was calculated.

### 3.6. Quantitative Real-Time PCR (qPCR)

Total RNA was extracted from cells using an RNA extraction kit (Accurate Biology, AG21023, Changsha, China) according to the manufacturer’s instructions. cDNA was synthesized using the All-in-one First Strand cDNA Synthesis SuperMix (Novoprotein Scientific Inc., E047-01A, Suzhou, China). qPCR was performed using SYBR qPCR SuperMix Plus (Novoprotein Scientific Inc., E096-01B, Suzhou, China) along with specific primers on a QuantStudio™ 6 Flex system (Applied Biosystems, Waltham, MA, USA). The thermal cycling conditions were as followed: 95 °C for 1 min, followed by 40 cycles of 95 °C for 20 s and 60 °C for 1 min. Relative mRNA levels were normalized to the internal reference gene *GAPDH*. The primer sequences are shown in Table 3.

### 3.7. Scratch Assay

Cells were seeded in 12-well plates and incubated overnight to reach 70–80% confluence. After stimulation with LPS for 24 h, a cross scratch was created in each well. Following washing with DPBS and replacement with fresh medium or administration, cells were photographed at 0 h and 24 h of culture. The scratch areas were quantified using ImageJ software (version 1.54), and the migration rate was calculated as: Migration rate (%) = 1 − (Blank areas 24 h/Blank areas 0 h).

### 3.8. Flow Cytometry

After a drug treatment, cells were harvested, washed with DPBS and counted. As a positive control, cells were heated in a 50 °C water bath for 5–10 min to induce apoptosis or necrosis. Cells were then resuspended in 100 μL of 1× Binding Buffer and stained with the FITC Annexin V Apoptosis Detection Kit for 15 min at room temperature. Subsequently, 400 μL of 1× Binding Buffer was added, and fluorescent signals were collected using a CytoFLEX flow cytometer (Beckman Coulter, Brea, CA, USA) under 4 °C and dark conditions. Annexin V-FITC and PI fluorescence were then analyzed. Compensation was performed using single-stained controls (Annexin V-FITC only and PI only) to correct for spectral overlap. Cells were initially identified using forward scatter area (FSC-A) versus side scatter area (SSC-A) to exclude debris and non-cellular events. A minimum of 10,000 events were acquired per sample.

### 3.9. Immunofluorescence

Cells were seeded onto cover slides and cultured overnight to reach 60–80% confluence. After LPS induction and treating with berberine or berberrubine, cells were fixed with 4% neutral formaldehyde for 15 min, permeabilized with DPBS containing 0.2% Triton X-100 for 5 min, and blocked with 10% BSA for 1–2 h. Subsequently, cells were incubated with primary antibodies and fluorescent secondary antibodies under dark conditions. The cell slides were mounted with anti-fade mounting medium and observed using a confocal fluorescence microscope (OLYMPUS, Tokyo, Japan).

### 3.10. Western Blot

Total proteins from cells or tissues were extracted using RIPA buffer supplemented with protease and phosphatase inhibitors (Biorigin, BN25015, Beijing, China). The lysates were centrifuged at 12,000 rpm for 15 min at 4 °C, and the supernatants were collected. Protein concentrations were determined using a BCA assay kit (LABLEAD, B5001, Beijing, China). After denaturation, samples were separated by SDS-PAGE and transferred onto nitrocellulose membranes (Sangon, F619512, Shanghai, China). The membranes were blocked with 5% non-fat milk or 5% BSA for 1.5 h at room temperature, followed by incubation with corresponding primary and secondary antibodies. Protein bands were visualized by enhanced chemiluminescence (NCM Biotech, P10100, Suzhou, China) using an imaging system (Bio-Rad, Hercules, CA, USA).

### 3.11. ELISA Assay

Serum levels of TNF-α and IL-6 were measured using commercial ELISA kits according to the manufacturer’s instructions. Briefly, standards and samples were added to 96-well plates and incubated with biotinylated antibodies and HRP conjugate at 37 °C. Color development was carried out using the substrate solution, and the absorbance was measured at 450 nm using a microplate reader after the reaction was terminated. Cytokine concentrations were calculated based on standard curves.

### 3.12. Molecular Docking

The structures of berberine (CID: 2353) and berberrubine (CID: 72703) were obtained from the PubChem database and converted to 3D format using Chem3D (version 22.0.0). The structure of human STAT3 (PDB ID: 6TLC) was retrieved from the RCSB, and water and solvent molecules were removed using PyMOL (version 3.1.6.1). Molecular docking within the box of the SH2 region of chain A was performed with AutoDock Vina (version 1.1.3). To ensure reproducibility, ten independent docking runs were performed. The docking poses with the lowest binding energies were selected and analyzed using PyMOL.

### 3.13. Molecular Dynamics

The AMBER family of protein force fields was applied for the protein, while the small molecules were parameterized following the GAFF protocol. The TIP4P-Ew water model was employed to describe solvent molecules. Each system was subjected to energy minimization, followed by NVT and NPT equilibration, prior to a 100 ns production run of molecular dynamics (MD). All MD simulations were performed using GROMACS (version 2026.0). Binding free energies were estimated via MM-PBSA calculations based on the Poisson–Boltzmann (PB) model, using the gmx_MMPBSA 1.6.4 tool. Polar close contacts were defined based on a distance cutoff of ≤3.5 Å between heavy atoms (N, O, or S). Van der Waals and close non-bonded contacts were evaluated collectively for heavy atoms within a distance range of 4.0–5.0 Å.

### 3.14. Mouse Intestinal Organoid Culture and Treatment

The intestines of C57BL/6 mice were dissected and flushed with cold PBS until the solution became clear. Tissues were dissociated with 5 mM EDTA at 4 °C for 30 min, followed by washing with cold PBS to remove EDTA. Crypts were released by mechanical trituration and then filtered to eliminate large debris. The collected suspension was subsequently centrifuged at 300× *g* for 3 min at 4 °C to pellet the crypts. The enriched crypts were resuspended in Matrigel (Mogengel Bio, 082703, Xiamen, China) and cultured in mouse intestine organoid medium (Mogengel Bio, MA-0817H006SP5) to allow proliferation and differentiation. To induce inflammation, organoids were first exposed to 100 μg/mL LPS for 24 h and then treated with 1 mM 5-ASA (Sigma-Aldrich, A79809), berberine (1× IC_50_ and 2× IC_50_), or berberrubine (1× IC_50_ and 2× IC_50_) for an additional 24 h. Subsequently, organoids were harvested, fixed, paraffin-embedded, sectioned, stained and observed using a confocal fluorescence microscope (OLYMPUS, Japan). Organoid budding was recorded using a brightfield microscopy. All experimental procedures and animal care were approved by the Experimental Animal Ethics Committee of Beijing University of Chinese Medicine (Approval No. BUCM- BUCM20260518-001).

### 3.15. Statistical Analysis

All data were expressed as mean ± standard deviation and all experiments had three biological replicates at least (*n* ≥ 3). Statistical analysis was performed using SPSS 20.0 software (IBM, Armonk, NY, USA). Differences between groups were analyzed by one-way analysis of variance (ANOVA) with LSD-t or Dunnett-t analysis. A value of *p* < 0.05 was considered statistically significant.

## 4. Conclusions

Together, our findings elucidate the molecular mechanism of berberine and berberrubine in treating IBD and demonstrate that their protective effects are mediated through STAT3 signaling. While previous studies have established the protective effects of berberrubine in DSS-induced colitis, our work identifies STAT3 as the critical mediator underlying these effects. The superior efficacy and favorable safety profile of berberrubine compared to berberine provide new insights for optimizing berberine-based therapy. Future studies using long-term colitis-associated cancer models and clinical samples will further help validate its therapeutic potential in inflammation-to-cancer transition. This work lays an experimental foundation for the further development of berberrubine as a potential therapeutic agent for IBD.

## Figures and Tables

**Table 1:** Cytotoxic effect of berberine and berberrubine in HCT-116 and NCM-460 cells.

Cell Line	Compound	IC_50_ (μM) *	
		WT	*STAT3* KO
HCT-116	berberine	12.26 ± 1.50	8.97 ± 2.40
	berberrubine	20.06 ± 1.67	23.78 ± 1.94
NCM-460	berberine	4.28 ± 2.32	1.33 ± 1.08
	berberrubine	40.67 ± 3.74	16.7 ± 5.65

* Data represent mean ± SD from three independent experiments.

**Table 2:** DAI score.

DAI Score	0	1	2	3	4
Weight loss	No weight loss	1–5%	5–10%	10–20%	>20%
Diarrhea	Normal	Loose stools	Mucus-like stool	Watery diarrhea	No stool
Hematochezia	Negative	+	2+	3+	4+

**Table 3:** Primer sequence.

Primer Name	Sequence (5′ to 3′)
*IL6* Forward	ACTCACCTCTTCAGAACGAATTG
*IL6* Reverse	CCATCTTTGGAAGGTTCAGGTTG
*TNF* Forward	CCTCTCTCTAATCAGCCCTCTG
*TNF* Reverse	GAGGACCTGGGAGTAGATGAG

## Data Availability

The data presented in this study are available from the corresponding author upon reasonable request.

## Supplementary Materials

The following supporting information can be downloaded at https://www.mdpi.com/article/10.3390/ijms27146341/s1.

## Abbreviations

The following abbreviations are used in this manuscript:

BBR	berberine
BRB	berberrubine
Cas9	CRISPR-associated protein 9
CD	Crohn’s disease
CRISPR	clustered regularly interspaced short palindromic repeats
DAI	disease activity index
DMSO	dimethyl sulfoxide
DSS	dextran sulfate sodium
ECM	extracellular matrix
ELISA	enzyme-linked immunosorbent assay
FAK	focal adhesion kinase
IBD	inflammatory bowel disease
IL-1β	interleukin-1β
IL-6	interleukin-6
KO	gene knock out
LPS	lipopolysaccharide
5-ASA	5-Aminosalicylic acid
MTT	methylthiazolyldiphenyl-tetrazolium bromide
MUC	mucins
P-gp	P-glycoprotein
PI	propidium iodide
qPCR	quantitative real-time PCR
SDS-PAGE	sodium dodecyl sulfate-polyacrylamide gel electrophoresis
sgRNA	single guide RNA
STAT3	signal transducer and activator of transcription 3
TCM	traditional Chinese medicine
TJ	tight junctions
TNF-α	tumor necrosis factor-α
UC	ulcerative colitis
WT	wild type
ZO-1	zonula occludens-1
