# Supplementary material for: Berberrubine, a Metabolite of Berberine, Attenuates Intestinal Barrier Dysfunction in Inflammatory Bowel Disease by Inhibiting STAT3
Source: Int J Mol Sci. 2026 Jul 16;27(14):6341. doi: 10.3390/ijms27146341 (PMC13409834; doi:10.3390/ijms27146341)

Supplementary Figures

Berberrubine, a metabolite of berberine, attenuates intestinal barrier dysfunction in inflammatory bowel disease by inhibiting STAT3

Figure legends:

S1. A-B. The survival rate (A) and body weight (B) of mice in each group. C-F. Protein expression and sequencing results of NCM-460 *STAT3* KO (C-D) and HCT-116 *STAT3* KO (E-F) cells. G. Positive control in flow cytometry experiments.

S2. A. The organoids were immunofluorescence stained with Muc2 and Lgr5. Scale bar: 50 μm. B. Morphology of mouse intestinal organoids. Scale bar: 100 μm.

S3. A. Bright-field images of intestinal organoids after 24 h of 100 μg/mL LPS stimulation. Scale bar: 100 μm. B. Bright-field images of intestinal organoids after LPS stimulation (Pre-treatment) and following 24 h treatment with 5 mM or 10 mM 5-ASA. Scale bar: 100 μm. C. Immunofluorescence staining of ZO-1 and Occludin in intestinal organoids following 24 h treatment with 5 mM 5-ASA after LPS stimulation. Scale bar: 50 μm.

S4. Molecular dynamics of STAT3 SH2 domain with berberrubine and berberine. A. number of hydrogen bonds across the simulation period. B. Solvent-accessible surface area (SASA). C. Free energy landscape (FEL). Blue and purple area indicate that the stable conformations of the complex are located within the minimum free energy region. D. Plot of the major residue energy decomposition from MM-PBSA.

S5. A. Growth inhibition curves of berberine and berberrubine in LS513. B. Western blot analysis of phosphorylated STAT3 in LS513 cells. C. Western blot analysis of phosphorylated FAK in NCM-460 after adding FAK inhibitor.

Supplementary Figure S1

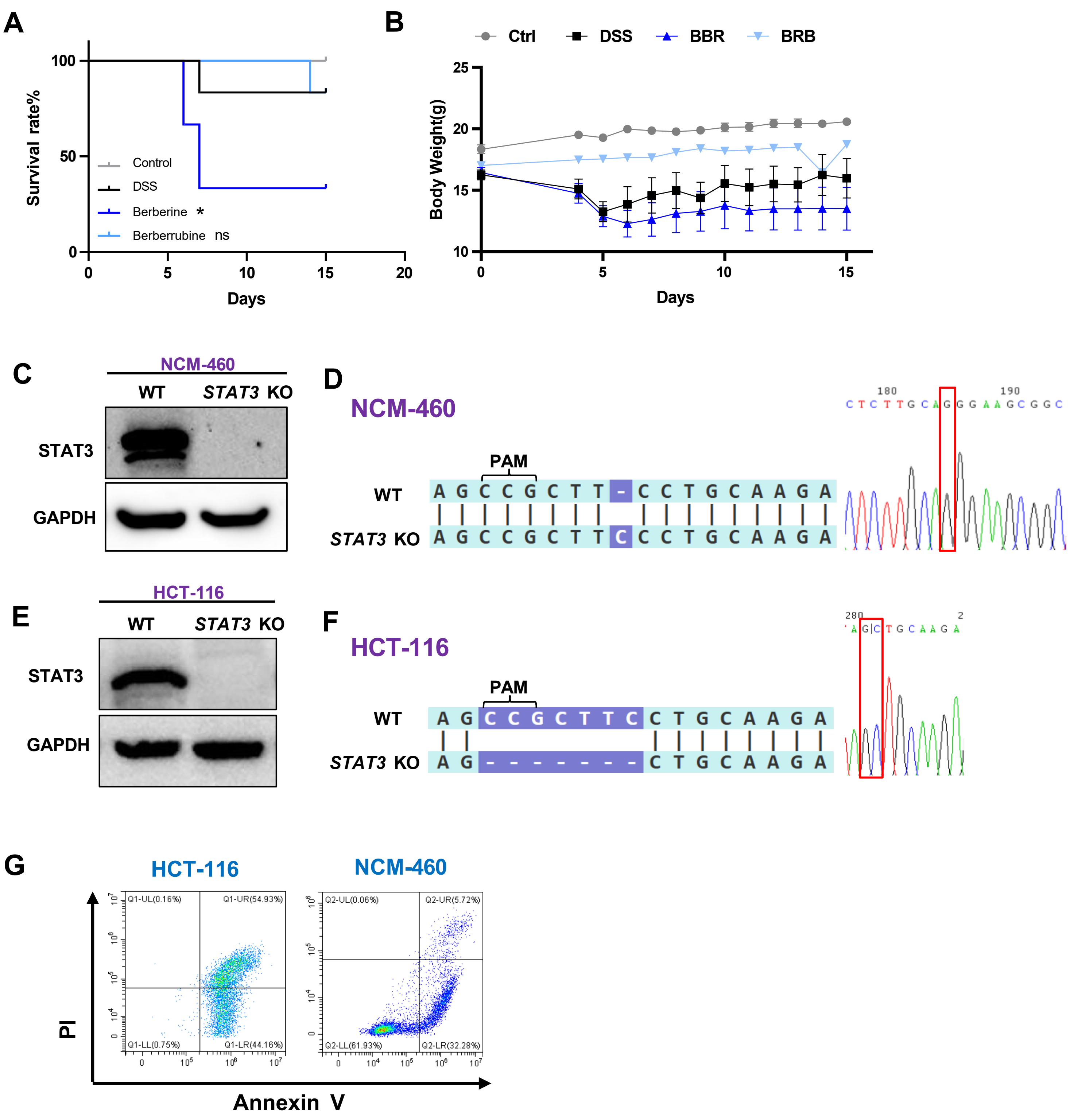

Supplementary Figure S2

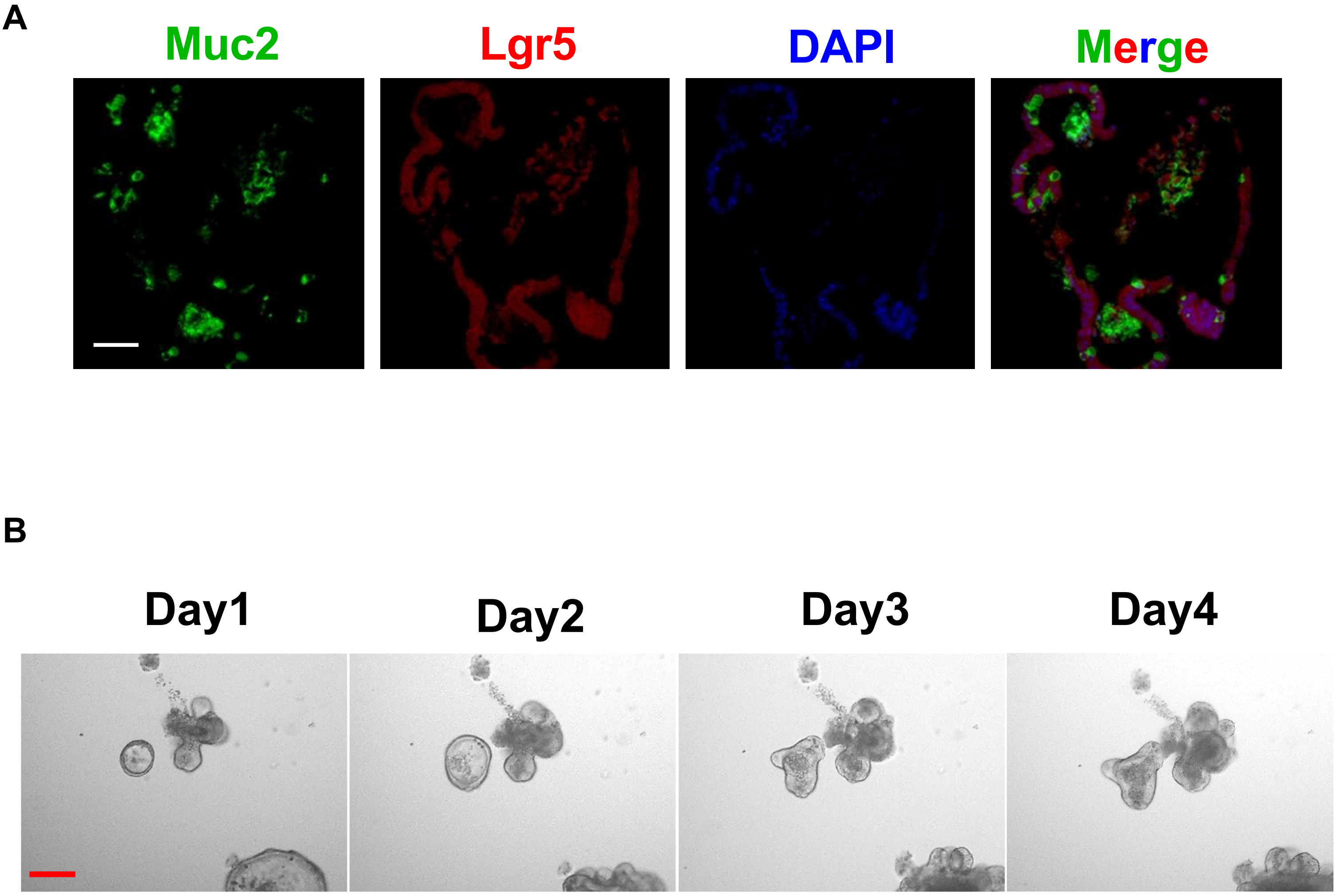

Supplementary Figure S3

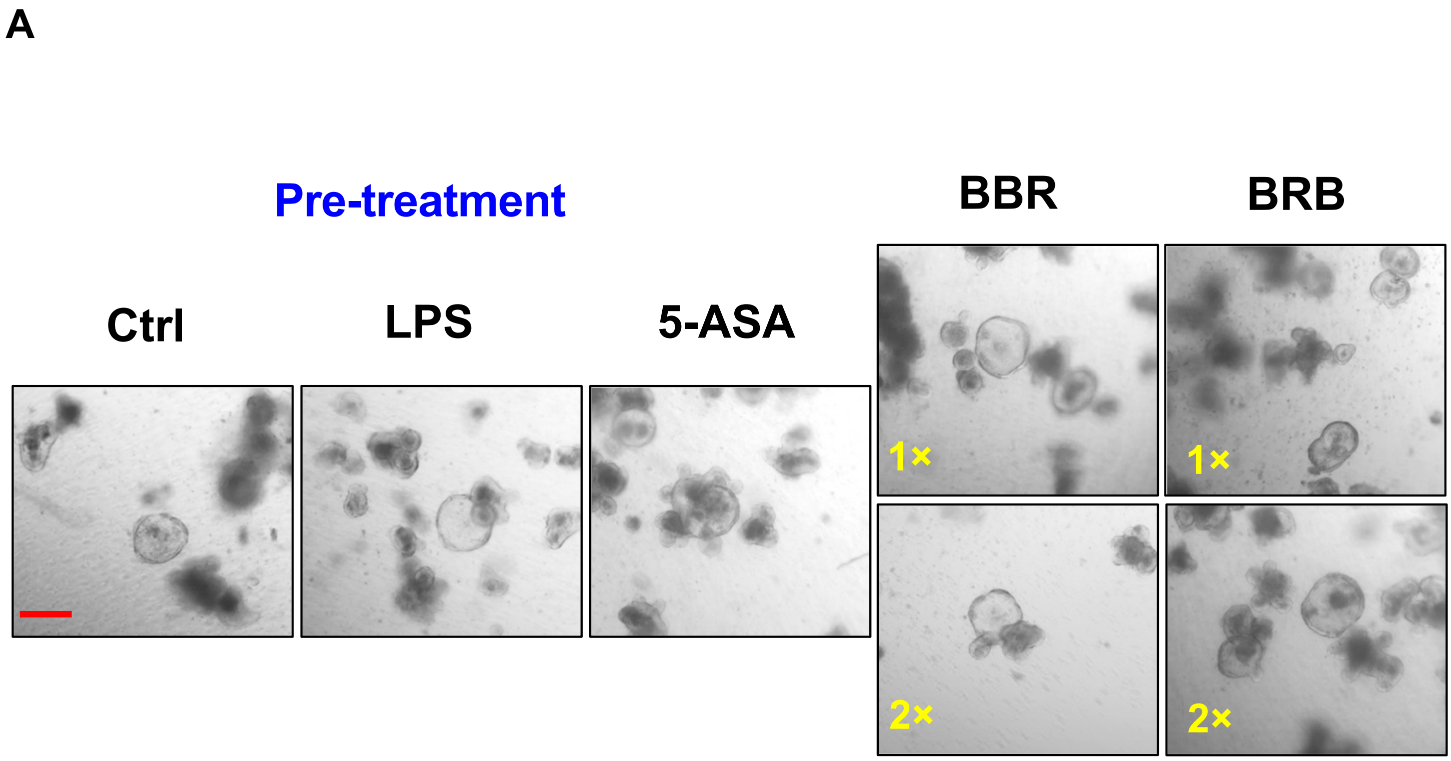

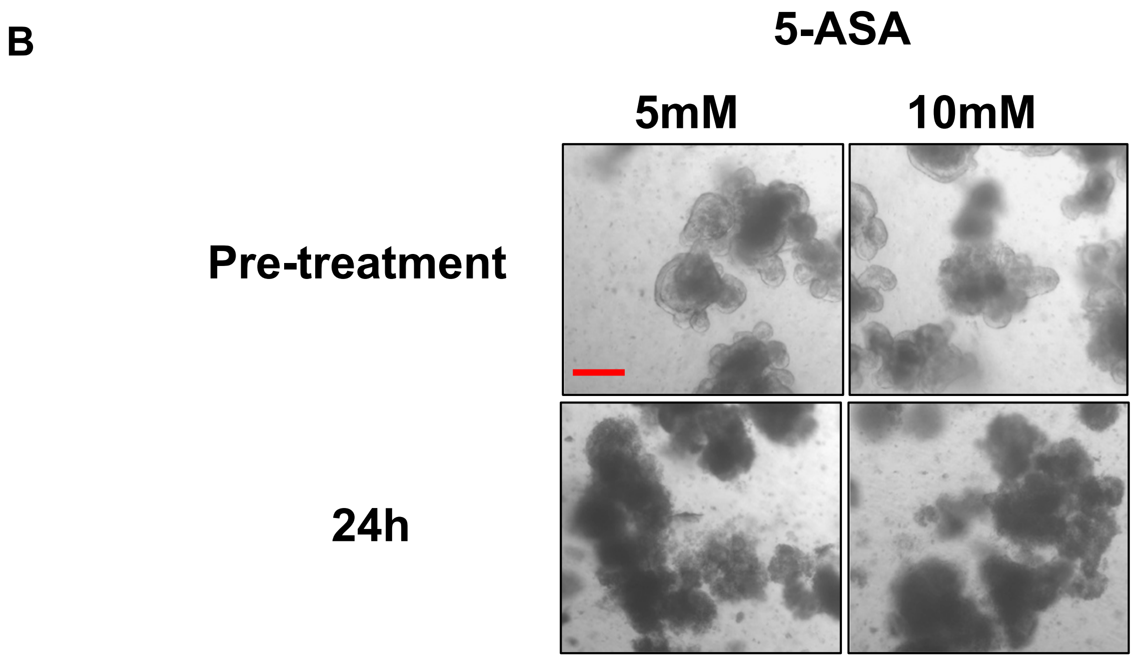

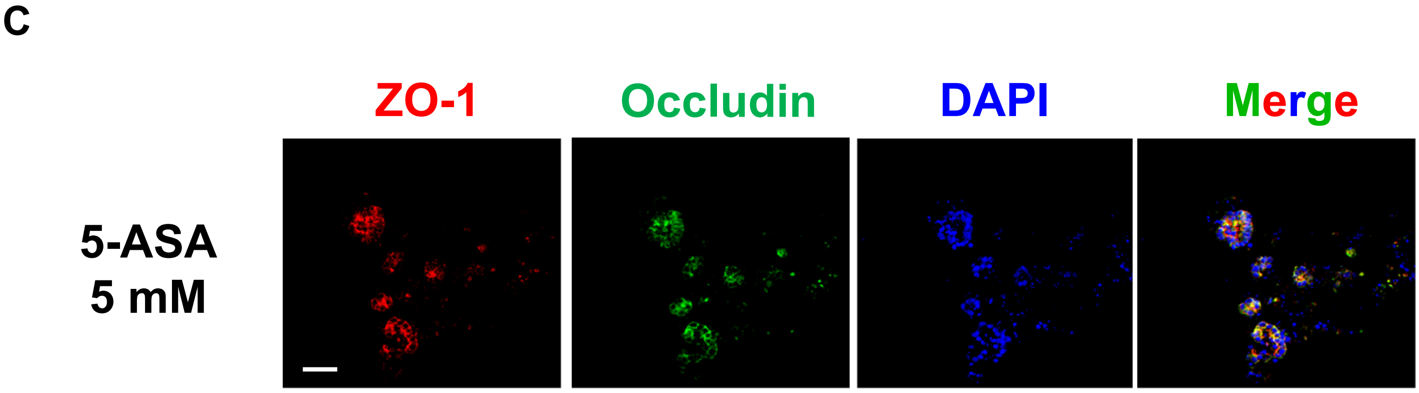

Supplementary Figure S4

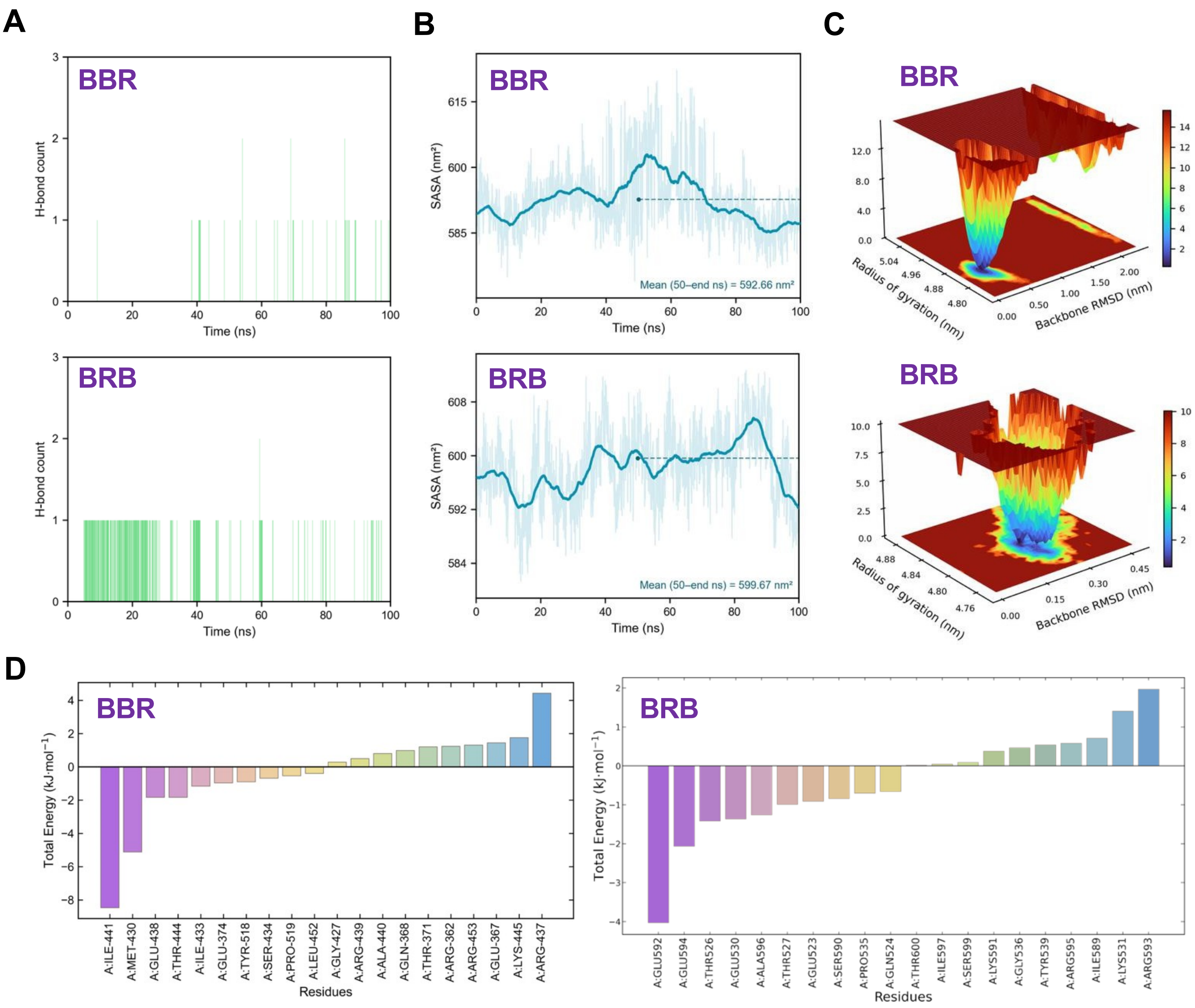

Supplementary Figure S5

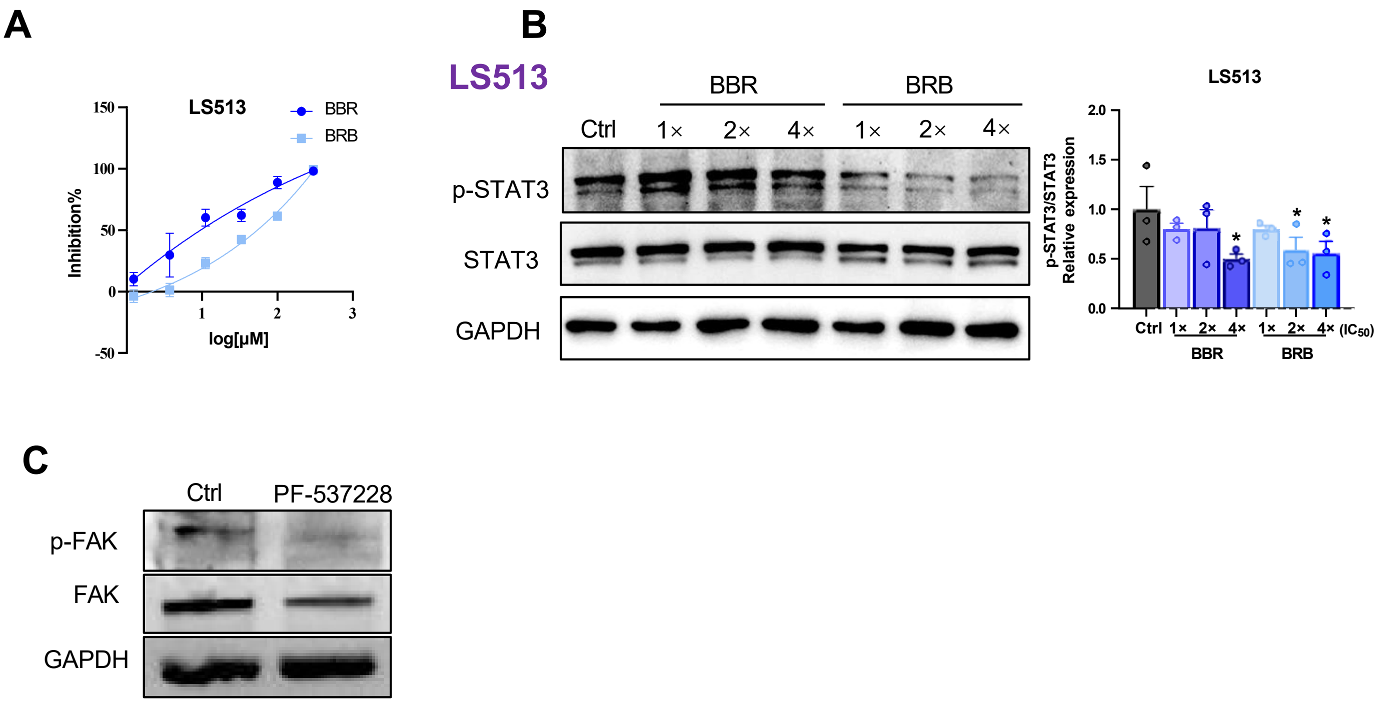

Supplement: Supplementary file 1 [file ijms-27-06341-s001.zip › ijms-4317485-supplementary.pdf]
